# A Study of* IL-1β*,* MMP-3*,* TGF-β1*, and* GDF5* Polymorphisms and Their Association with Primary Frozen Shoulder in a Chinese Han Population

**DOI:** 10.1155/2017/3681645

**Published:** 2017-06-06

**Authors:** Wenxiang Chen, Jia Meng, Hong Qian, Zhantao Deng, Shuo Chen, Haidong Xu, Wenshuang Sun, Yiying Wang, Jianning Zhao, Nirong Bao

**Affiliations:** Department of Orthopedics, Jinling Hospital, Southern Medical University, 305 Zhongshan East Road, Nanjing, Jiangsu 210002, China

## Abstract

Primary frozen shoulder (PFS) is a common condition of uncertain etiology that is characterized by shoulder pain and restriction of active and passive glenohumeral motions. The pathophysiology involves chronic inflammation and fibrosis of the joint capsule. Single nucleotide polymorphisms (SNPs) at* IL-1β*,* MMP3*,* TGF-β1*, and* GDF5* have been associated with risk of a variety of inflammatory diseases; however, no studies have examined these SNPs with susceptibility to PFS. We investigated allele and genotype frequencies of rs1143627 at* IL-1β*, rs650108 at* MMP-3*, rs1800469 at* TGF-β1*, and rs143383 at* GDF5* in 42 patients with PFS and 50 healthy controls in a Chinese Han population. Serum samples from both cohorts were evaluated to determine the expression levels of IL-1*β*. We found that the* IL-1β* rs1143627 CC genotype was associated with a decreased risk of PFS compared to the TT genotype (*P* = 0.022) and that serum IL-1*β* was expressed at a significantly higher level in the PFS cohort compared to that found in the control group (*P* < 0.001). Our findings indicated no evidence of an association between rs650108, rs1800469, or rs143383 and PFS. IL-1*β* is associated with susceptibility to PFS and may have a role in its pathogenesis in a Chinese Han population.

## 1. Introduction

Primary frozen shoulder (PFS) is a common condition of uncertain etiology with a prevalence of 2% to 5% [1], which is characterized by shoulder pain and restriction of active and passive glenohumeral motions. The pathophysiology of PFS involves chronic inflammation and fibrosis of the joint capsule [2]. Zuckerman and Rokito [3] proposed the classification of frozen shoulder into primary and secondary where secondary frozen shoulder includes all cases in which the underlying etiology or associated condition can be identified. PFS is a painful contracture of the glenohumeral joint that arises spontaneously without a preceding event [4]. The severe pain and stiffness of PFS interfere with activities of daily living and the ability to perform at work. PFS has traditionally been described having three phases, specifically, freezing, the frozen condition, and thawing. The duration may be from 1 to 3 years, and the condition can be self-limited [5]. Initially, PFS is characterized by a gradual onset of pain that is most symptomatic at night. This freezing stage lasts from a few weeks to a few months, which is followed by a stiff or frozen phase that is the consequence of pain relief of immobility that lasts for 4–12 months. Finally, this is followed by the thawing phase that may have a subjective relief over several months. Hand et al. [6] reported that PFS occurs more often in females compared to males, and it is a condition of middle age affecting individuals of 40–60 years old, which is within the working-age range and, therefore, has a significant economic impact on both individuals and society.

Cytokines exert a pivotal role in disease development and may act as potential diagnostic markers of inflammatory disorders, such as PFS. Among the different cytokines, the proinflammatory cytokine interleukin-1 (IL-1) has been implicated in both acute and chronic inflammatory diseases. The master cytokine IL-1 has a role in essential functions such as immune cell recruitment, cell proliferation, tissue destruction, and vascular smooth muscle cell contraction [7]. It has been reported that inflammatory mediators such as IL-1*α*, IL-1*β*, IL-6, and tumor necrosis factor alpha (TNF-*α*) have significant roles in inflammation and the collagen catabolic process [7, 8]. Rodeo et al. [9] demonstrated that a variety of inflammatory cytokines such as IL-1*α*, IL-1*β*, IL-6, and TNF-*α* are found in the synovium of the glenohumeral joint of patients with PFS. In addition, researchers from a prospective randomized study reported that IL-1*α*, IL-1*β*, and TNF-*α* were expressed at significantly higher levels in the joint capsules of individuals from the PFS group compared to that found in the control group [10]. In addition, recent studies have shown that overexpression of inflammatory cytokines in the glenohumeral joint capsule has an essential role in the pathogenesis of PFS [11, 12].

IL-1 induces the expression of matrix metalloproteinases (MMPs), including MMP-1, MMP-3, and MMP-13 [8, 13, 14]. MMPs are zinc-dependent proteinases that degrade the connective-tissue matrix and, more specifically, the extracellular matrix [15], whereas tissue inhibitor of metalloproteinases (TIMPs) are MMP inhibitors that regulate the synthesis and activity of MMPs. MMP-3, which is an essential member of the MMP family, is produced by various types of cells including fibroblasts, smooth muscle cells, and macrophages [16, 17]. Previous studies have reported that fibroblasts and myofibroblasts are the predominant cells in the shoulder joint capsule, laying down a dense type III collagen matrix, which underlies the frozen condition of PFS [18]. Kabbabe et al. [11] detected that the fibrogenic cytokine MMP-3 was elevated in patients with PFS compared to controls. Similarly, A. M. T. Lubis and V. K. Lubis [19] compared the serum levels of proteins related to PFS, such as TGF-*β*, MMPs, and TIMPs between patients with PFS and normal individuals and found that baseline levels of MMPs were significantly lower, and levels of TIMPs and TGF-*β*1 were significantly higher, in patients with PFS than in controls. Hutchinson et al. [20] postulated that the development of PFS was because of a decrease in the MMP : TIMP ratio, which leads to increased synthesis and deposition of collagen.

Transforming growth factor-*β*1 (TGF-*β*1) is a multifunctional cytokine that modulates cell growth, differentiation, and matrix production [21, 22]. Moreover, TGF-*β*1 is a profibrotic factor that can regulate expression of MMPs and TIMPs and the signaling pathways involved in tissue fibrosis [23]. Growth differentiation factor 5 (GDF5), which is also known as cartilage-derived morphogenetic protein 1 (CDMP1) or bone morphogenetic protein 14 (BMP14), belongs to the TGF-*β* superfamily [24, 25]. Increased levels of GDF-5 stimulate the production of type I collagen subunit genes by modulating integrin TNF-*α*2 activity [26]. GDF5 is involved in musculoskeletal processes and affects synovial joint and tendon formation. A. M. T. Lubis and V. K. Lubis [19] detected significantly increased TGF-*β* in patients with PFS compared to that found in controls. In addition, Rodeo et al. [9] previously reported an increase of TGF-*β*1 positive staining in PFS tissue samples.

Single nucleotide polymorphisms (SNPs) at* IL-1β*,* MMP3*,* TGF-β1*, and* GDF5* have been associated with risk of a wide variety of diseases, such as keratoconus, chronic periodontitis, and lumbar disc degeneration [27–29]. Currently, the etiology and pathogenesis of PFS are not fully understood, and, therefore, research towards identifying culprit genes is warranted. Furthermore, few studies have evaluated the contributions of specific SNPs at candidate loci with PFS onset. To identify the effect of specific candidate gene polymorphisms on the pathogenesis of PFS, we genotyped polymorphisms at* IL-1β*,* MMP-3*,* TGF-β1*, and* GDF5* to determine whether there is evidence of an association in a Chinese Han population. Additionally, we investigated whether there was an association between serum IL-1*β* expression and PFS.

## 2. Materials and Methods 

### 2.1. Participants

Our study was approved by the institutional review boards of the hospital, and informed consent was obtained from all study participants according to the Helsinki Declaration. Forty-two patients with PFS were recruited from the Department of Orthopedics, at the Nanjing Jinling Hospital, between January 2016 and July 2016. All recruited patients came to the hospital for treatment of PFS and were examined and diagnosed by orthopedic specialists at the department. Inclusion criteria for participation in our study were as follows: (1) shoulder pain for at least 1 month, (2) global restriction of passive shoulder motion with normal findings on plain radiographs, (3) thickening of the joint capsule particularly in the axillary region and thickening of the coracohumeral ligament with no pathological findings related to the rotator cuff, labrum, long head of the biceps, or acromioclavicular joint on magnetic resonance imaging (MRI) [30], and (4) no risk factors such as diabetes, cardiovascular disease, or thyroid disease. In addition, the following exclusion criteria were used: (1) rotator cuff disorder (i.e., tendinitis and partial-thickness or full-thickness tears), (2) biceps tendinitis or calcific tendinitis, (3) previous ipsilateral breast surgery, cervical radiculopathy, or chest wall tumor, (4) previous cerebrovascular accident, (5) previous humeral shaft fracture, scapulothoracic abnormalities, acromioclavicular arthritis, or clavicle fracture, (6) being diagnosed with diabetes mellitus, hyperthyroidism, or hypothyroidism, and (7) a present history of clinically confirmed any other disease causing the increased levels of IL-1*β*. Fifty healthy controls were recruited from individuals at the hospital for general health exams. The control group was composed of individuals without any history of PFS and adhered to the exclusion criteria as determined by physical examination and laboratory findings. All the subjects of the case group were in the freezing stage, characterized by a gradual onset of pain that is most symptomatic at night.

### 2.2. DNA Extraction and Genotyping

Peripheral blood samples were collected from all 92 participants in our study by standard venipuncture in tubes containing ethylenediaminetetraacetic acid (EDTA) at seven in the morning, which were stored at −70°C until DNA extraction. DNA was extracted from white blood cell fractions using the Chelex-100 extraction method. Purity and concentration of the extracted DNA were determined spectrophotometrically. Extracted DNA was stored at −20°C for further analysis. SNP genotyping was performed on an Agena Bioscience MassARRAY® platform utilizing the iPLEX® assay (Agena Bioscience Inc., San Diego, CA, USA). PCR primers and single base pair extension primers were designed using ADS 2.0 [13] and are provided in Table 1. Polymerase chain reaction (PCR) amplification and extension reactions were performed according to the manufacturer's instructions (iPLEX Gold, Agena Bioscience Inc.). Extension product sizes were determined by mass spectrometry using the MassARRAY system (Agena Bioscience Inc.). Samples were run in duplicate and positive and negative control samples were included in each plate to assess genotyping accuracy.

### 2.3. Enzyme-Linked Immunosorbent Assay

IL-1*β* levels in serum samples from participants were determined using a commercially available enzyme-linked immunosorbent assay (ELISA) kit (AMEKO Inc., Shanghai, China), which was performed according to the manufacturer's instructions. Briefly, serum samples were diluted 1 : 10,000 in Tris-buffered saline solution (AMEKO Inc.) containing 1% bovine serum albumin and 0.5% Tween-20 and were incubated overnight in plates at 4°C, after which plates (AMEKO Inc.) were washed 5 times. IL-1*β* was added to each plate in a series dilution, and a standard curve was constructed from which the concentrations of IL-1*β* were obtained. Absorbance values were determined using an ELISA microplate reader at 450 nm. Each sample was tested in duplicate.

### 2.4. Statistics

The statistical software package SPSS v21.0 (SPSS Inc., Chicago, IL, USA) was used for statistical analysis. The chi-square test and Student's *t*-test were performed to analyze demographic data between patient and control cohorts, whereas the expression levels of IL-1*β* in the two groups were compared using Student's *t*-test. We used the chi-square test and Fisher's exact test for comparisons of allele and genotype frequencies between patients with PFS and controls. Genotype and allele frequencies were evaluated for Hardy-Weinberg equilibrium (HWE) using the chi-square test. Odds ratios (ORs) and 95% confidence intervals (CIs) were calculated. All statistical tests were two-tailed, and *P* values < 0.05 were considered significant.

## 3. Results

### 3.1. Characteristics of Study Population

Forty-two patients with PFS and fifty healthy controls were enrolled in our study, the characteristics of which are summarized in Table 2. We found that the mean age and standard deviation (SD) of the control group and patient group was 53.56 ± 1.80 years and 52.76 ± 2.77 years, respectively. In addition, the male/female ratio was 20/22 and 23/27 in patients and controls, respectively, and the mean BMI of patients with PFS was 23.5 ± 1.00 kg/m^2^ compared to that of 23.30 ± 1.13 kg/m^2^ in controls. We found no significant differences in the mean age, gender ratio, and BMI between our two cohorts.

### 3.2. Genotype and Allele Distributions of IL-1*β*, MMP-3 TGF-*β*1, and GDF5 Polymorphisms

All polymorphisms tested were in HWE in both of our cohorts (all *P* > 0.05). We found a significant difference in* IL-1β* rs1143627 genotype frequencies between patients with PFS and controls (*P* = 0.020). As shown in Table 3, the CC genotype of* IL-1β* rs1143627 was associated with a statistically significant decreased risk of PFS compared to individuals with the TT genotype (OR = 0.17, 95% CI = 0.03–0.87, *P* = 0.022). However, we found that individuals with a TC genotype were not at an increased risk of susceptibility to PFS compared to individuals with the TT genotype. We found no significant differences in the allele and genotype frequencies of* MMP-3* rs650108 (*P* = 0.900 and *P* = 0.930),* TGF-β1* rs1800469 (*P* = 0.872 and *P* = 0.799), and* GDF5* rs143383 (*P* = 0.828 and *P* = 0.790) between our two cohorts and found no significant association for PFS risk in all tested models with these three polymorphisms (Table 3).

### 3.3. IL-1*β* Production Is Increased in the Serum of Patients with PFS

IL-1*β* level was 28.55 ± 2.58 pg/mL in patients with PFS compared to 20.12 ± 2.28 pg/mL in controls (Table 4). We found that this increased level of serum IL-1*β* expression in patients was significantly higher than that found in the control group (*P* < 0.001).

## 4. Discussion

In the present study, our findings demonstrated that patients with the rs1143627 CC genotype at* IL-1β* have a significantly decreased risk of PFS, whereas the frequencies of patients with the TT or TC genotype were not significantly different from that found in healthy controls in a Chinese Han population. In contrast, we found no significant evidence of an association between rs650108 at* MMP-3*, rs1800469 at* TGF-β1*, and rs143383 at* GDF5* and PFS susceptibility in the same cohort. To date, studies examining the etiology and pathogenesis of PFS as well as efforts to identify culprit genes have been rarely performed. In fact, no previous studies have reported an association between polymorphisms at* IL-1β*,* MMP-3*,* TGF-β1*, and* GDF5* and susceptibility to PFS.

To determine whether there was a significant difference in serum IL-1*β* expression in samples from our two cohorts, we measured expression levels of IL-1*β* using ELISA and found a significantly increased level of IL-1*β* in patients with PFS compared to that in controls (28.55 ± 2.58 pg/mL versus 20.12 ± 2.28 pg/mL), a finding indicative of the upregulation of IL-1*β* expression in affected individuals (*P* < 0.001). Rodeo et al. [9] and Lho et al. [10] independently reported increased expression of IL-1*β* in the synovium of the shoulder and in the joint capsules in patients with PFS compared to that in controls. In fact, both studies and the findings from our study indicate that increased levels of IL-1*β* are associated with risk of PFS and that this increased expression is consistently found in serum as well as the synovium and joint capsules of the shoulder in individuals with PFS recruited in three different cohorts.

Because of this lack of prior genetic investigation in PFS, we identified the polymorphisms tested in this study in candidate genes (*IL-1β*,* MMP-3*,* TGF-β1*, and* GDF5*). Polymorphisms in these genes were associated with susceptibility to other human diseases. The IL-1 gene cluster is located on chromosome 2q13–2q14.1 and is comprised of nine genes:* IL-1α*,* IL-1β*, the IL-1 receptor antagonist* (IL-1RN)*, and six IL-1-like factor genes (*IL-1F5* through* IL-1F10*) [31].* IL-1β* is in a 70–110 kb region of chromosome 2q13–21 and consists of 7 exons and 6 introns [32]. At least 20 SNPs have been reported in the region of* IL-1β*, among which rs1143627 was identified as an important functional polymorphism that could influence gene transcription and, therefore, lead to functional changes [33]. A case-control study of 232 patients with preeclampsia and 447 healthy controls showed that* IL-1β* rs1143627 was associated with risk of preeclampsia in a Chinese Han population [34]. This study shares similarities with our current study. First, all subjects in the two studies were from a Chinese Han population. Second, the results of both studies showed evidence of an association between* IL-1β* rs1143627 and specific diseases. Third, both studies used the chi-square test to evaluate differences in allele and genotype frequencies between cases and controls. Furthermore, there is evidence that this polymorphism may have a role in susceptibility to other diseases in different ethnic groups. A case-control study of 93 patients with papillary thyroid carcinoma (PTC) and 324 controls found that* IL-1β* rs1143627 may be associated with risk of PTC in a Korean population [35]. Similarly, another study found that rs1143627 genotypes may be associated with low-dose aspirin-induced peptic ulcers in a Korean ethnic group [36].

A Chinese research [37] suggested that the MMP3 rs650108 polymorphism was significantly associated with susceptibility of frozen shoulder. However, subjects of this study were patients with frozen shoulder rather than with primary frozen shoulder. Frozen shoulder involves both primary frozen shoulder and secondary frozen shoulder. Otherwise, all individuals of this study were from north China, while ours were from south China. And the result could be influenced by different regions. A previous study [28] reported an association between the* MMP-3* rs650108 polymorphism and chronic periodontitis in a Brazilian population. Similarly, Raleigh et al. [16] investigated whether variants within* MMP-3* contributed to Achilles tendinopathy in a Caucasian population and found that* MMP-3* rs650108 was associated with increased risk of Achilles tendinopathy.* TGF-β1* is located on chromosome 19q13.2 [38]. A study [39] found that rs1800469 at* TGF-β1* was associated with end stage renal disease in individuals from the Jammu and Kashmir regions in Northern India, while a study [29] of women from Northern Europe found an association between lumbar disc degeneration and the rs143383 polymorphism at* GDF*5, which indicates that* GDF5* is a culprit gene for lumbar disc degeneration in that population. Although these studies show a significant contributory role of these polymorphisms at* MMP-3*,* TGF-β1*, and* GDF5* in risk of a variety of diseases, none of the three polymorphisms tested in this study was associated with susceptibility to PFS in our Chinese Han cohort. One reason that may account for these discrepancies is that the individuals in these studies were from different races and ethnic populations, including being Brazilian, being Caucasian, individuals from the Jammu and Kashmir regions from Northern India, women from Northern Europe, and being Chinese Han, and that ethnicity can significantly influence genotype frequency. In addition, these studies examined the pathogenesis of different diseases, which may have different underlying etiologies as well as significantly distinct occurrence and progression within different populations [16, 28, 29, 39].

Currently, research in PFS has mainly focused on pathology, imaging studies, clinical diagnosis, and treatment. In contrast, determining the underlying pathogenesis of the condition has been significantly underinvestigated with previous studies dominantly focusing on the role of inflammation and fibrosis in the occurrence and development of PFS. While these studies confer a partial understanding of the cause of the disease, our study was the first to investigate the putative role of* IL-1β* rs1143627 with susceptibility to PFS and its effect on IL-1*β* expression in affected individuals from a Chinese Han population and, therefore, contributes to an improved understanding of the underlying genetic etiology and pathogenesis of the condition. Furthermore, our study will be helpful to clinical practice. On the one hand, the clinical significance of the current results indicates that IL-1*β* level of the subjects of the case group was significantly higher than that of the control group. We can examine IL-1*β* level of the patient who comes to hospital to seek medical help, which is regarded as a biomarker when doctors diagnose PFS in clinical practice. On the other hand, further study investigating how* IL-1β* rs1143627 CC genotype decreases the risk of PFS compared to* IL-1β* rs1143627 TT genotype is warranted on the basis of our preliminary research. Once we find the specific cause, we may implement PFS prevention by medical intervention directed at individuals.

However, there are limitations to our study. First, the sample size of our affected cohort is relatively small so it may be underpowered to identify small or modest effects of association between* MMP-3* rs650108,* TGF-β1* rs1800469, and* GDF5* rs143383 with PFS. Therefore, future studies using a larger number of samples are warranted. Second, the participants in our study are specifically from a Chinese Han ethnic group, and it is possible that the underlying genetic etiology in Chinese Han may not involve these candidate genes, which may have a role in other ethnic populations. Thus, it would be informative to test these variants in different populations for a more comprehensive assessment of their prospective role in PFS. Third, we investigated four genes for evidence of susceptibility to PFS, and it is possible that SNPs in other candidate genes that have yet to be tested are associated with disease risk in Chinese Han. Otherwise, DNA could also be extracted from tissue of the capsule from the shoulder joint of participants. Our study has several advantages. The robustness of our genotyping results was guaranteed by strict laboratory operation. In addition, SNP genotyping was performed on the MassARRAY platform, which was not only convenient but also highly accurate. Furthermore, we performed venipuncture on the participants at the same time in the morning as we cannot exclude the possibility of circadian variation in IL-1*β* level. Finally, recruited individuals from affected and healthy control groups were randomly selected and the SNPs that were investigated were in HWE in both cohorts.

## 5. Conclusions

Our findings of an association between the* IL-1β* rs1143627 polymorphism and risk of PFS and evidence of increased IL-1*β* in the serum of affected individuals in our cohort of Chinese Han contribute to an improved understanding of the pathogenesis of the disease at the molecular level and may provide novel insights for the diagnosis and treatment of PFS at an early stage. To better elucidate the functional roles of the polymorphism in the etiology of PFS, in-depth molecular studies are required to clarify the biological signaling mechanisms involved and to determine the exact effects of the* IL-1β* rs1143627 polymorphism in regulating IL-1*β* expression in the Chinese Han population.

## Figures and Tables

**Table 1:** PCR primers and single base pair (bp) extension primers of 4 SNPs at *IL-1β*,* GDF5*, *MMP-3*, and *TGF-β1*.

Genes	SNPs	PCR primer sequence (5′→3′)	Single bp extension primer sequence
*IL-1β*	rs1143627-F	ACGTTGGATGTTGTGCCTCGAAGAGGTTTG	TCCCTCGCTGTTTTTAT
	rs1143627-R	ACGTTGGATGTCTCAGCCTCCTACTTCTGC	
*MMP-3*	rs650108-F	ACGTTGGATGTCAGGTAGAGGTGACAAGTG	CCAGTGGGTGAGGTTAGA
	rs650108-R	ACGTTGGATGGTCACTGTCTCATTGTGTGT	
*TGF-β1*	rs1800469-F	ACGTTGGATGTACAGGTGTCTGCCTCCTGA	ACTCCTGACCCTTCCATCC
	rs1800469-R	ACGTTGGATGAAGAGGGTCTGTCAACATGG	
*GDF5*	rs143383-F	ACGTTGGATGCAGCAGCTGAAAATAACTCG	TCTTGAAAGGAGAAAGCC
	rs143383-R	ACGTTGGATGTTCAAGCCCTCAGTCAGTTG	

PCR: polymerase chain reaction; SNP: single nucleotide polymorphism.

**Table 2:** Characteristics of the study population in patients with PFS and controls.

Characteristics	Cases (*n* = 42)	Controls (*n* = 50)	*P* value
Male/female	20/22	23/27	0.877
Age (years)	53.86 ± 1.60	54.80 ± 2.58	0.175
BMI (kg/m^2^)	23.15 ± 1.00	23.31 ± 1.14	0.481

BMI: body mass index; PFS: primary frozen shoulder.

**Table 3:** Comparisons of genotype and allele distributions of* IL-1β*,* MMP-3*, *TGF-β1*, and *GDF5* polymorphisms between patients with PFS and controls.

SNP	Cases	Controls	OR (95% CI)	*P* value
	*n* (%)	*n* (%)		
*IL-1β* rs1143627				
Genotype				
TT	14 (33.3)	15 (30.0)	1.00^a^	
TC	26 (61.9)	22 (44.0)	1.27 (0.50–3.19)	0.616
CC	2 (4.8)	13 (26.0)	0.17 (0.03–0.87)	0.022
TC + CC	28 (66.7)	35 (35.0)	0.86 (0.36–2.07)	0.732
Allele				
T	54 (64.3)	52 (52.0)	1.00^a^	
C	30 (35.7)	48 (48.0)	0.60 (0.33–1.09)	0.093
*MMP-3* rs650108				
Genotype				
AA	18 (42.9)	20 (40.0)	1.00^a^	
AG	16 (48.1)	21 (42.0)	0.85 (0.34–2.10)	0.720
GG	8 (19.0)	9 (18.0)	0.99 (0.34–3.11)	0.983
AG + GG	24 (67.1)	30 (60.0)	0.89 (0.39–2.04)	0.782
Allele				
A	52 (61.9)	61 (61.0)	1.00^a^	
G	32 (38.1)	39 (39.0)	0.96 (0.53–1.75)	0.900
*TGF-β1* rs1800469				
Genotype				
CC	9 (21.4)	13 (26.0)	1.00^a^	
CT	23 (54.8)	24 (48.0)	1.38 (0.50–0.39)	0.533
TT	10 (23.8)	13 (26.0)	1.11 (0.34–3.63)	0.862
CT + TT	33 (78.6)	37 (74.0)	1.29 (0.49–3.40)	0.609
Allele				
C	41 (48.9)	50 (50.0)	1.00^a^	
T	43 (51.1)	50 (50.0)	1.05 (0.59–1.87)	0.872
*GDF5* rs143383				
Genotype				
TT	22 (52.4)	24 (48.0)	1.00^a^	
TC	18 (42.9)	22 (44.0)	0.89 (0.38–2.09)	0.793
CC	2 (4.8)	4 (8.0)	1.54 (0.42–5.64)	0.513
TC + CC	20 (47.7)	26 (52.0)	0.84 (0.37–1.91)	0.675
Allele				
T	62 (73.8)	70 (70.0)	1.00^a^	
C	22 (26.2)	30 (30.0)	1.07 (0.58–1.98)	0.828

^a^Reference genotype/allele; CI: confidence interval; OR: odds ratio; PFS: primary frozen shoulder; SNP: single nucleotide polymorphism.

**Table 4:** Comparison between serum IL-1*β* level in patients with PFS and controls.

Group	*n*	Levels of IL-1*β* (pg/mL)	*P* value
Cases	42	28.55 ± 2.58	<0.001
Controls	50	20.12 ± 2.28	

PFS: primary frozen shoulder.
